# [^18^F]FDOPA PET/CT is superior to [^68^Ga]DOTATOC PET/CT in diagnostic imaging of pheochromocytoma

**DOI:** 10.1186/s13550-023-01056-4

**Published:** 2023-12-18

**Authors:** Peter Iversen, Stine Kramer, Andreas Ebbehoj, Esben Søndergaard, Kirstine Stochholm, Per Løgstrup Poulsen, Karin Hjorthaug

**Affiliations:** 1 Department of Nuclear Medicine and PET Centre, Aarhus University Hospital, 8200 Aarhus N, Denmark; 2 Department of Endocrinology and Internal Medicine, Aarhus University Hospital, 8200 Aarhus N, Denmark

**Keywords:** FDOPA, DOTATOC, Pheochromocytoma, Paraganglioma, PET, PPGL, PCC, PC, PGL

## Abstract

**Background:**

Both [^18^F]FDOPA (FDOPA) and [^68^Ga]DOTATOC PET/CT (DOTATOC) are widely used for detection of pheochromocytomas/paraganglioma (PPGL). However, direct comparisons of the performance of the two tracers are only available in small series. We conducted a retrospective comparative analysis of FDOPA and DOTATOC to assess their sensitivity and accuracy in detecting PPGL when administered based on suspicion of PPGL. We consecutively included patients referred on suspicion of PPGL or PPGL recurrence who were scanned with both FDOPA and DOTATOC. Both scans were reviewed retrospectively by two experienced observers, who were blinded to the final diagnosis. The assessment was made both visually and quantitatively. The final diagnosis was primarily based on pathology.

**Results:**

In total, 113 patients were included (97 suspected of primary PPGL and 16 suspected of recurrence). Of the 97 patients, 51 had pheochromocytomas (PCC) (in total 55 lesions) and 6 had paragangliomas (PGL) (in total 7 lesions). FDOPA detected and correctly localized all 55 PCC, while DOTATOC only detected 25 (sensitivity 100% vs. 49%, *p* < 0.0001; specificity 95% vs. 98%, *p* = 1.00). The negative predictive value (100% vs. 63%, *p* < 0.001) and diagnostic accuracy (98% vs. 70%, *p* < 0.01) were higher for FDOPA compared to DOTATOC. FDOPA identified 6 of 6 patients with hormone producing PGL, of which one was negative on DOTATOC. Diagnostic performances of FDOPA and DOTATOC were similar in the 16 patients with previous PPGL suspected of recurrence.

**Conclusions:**

FDOPA is superior to DOTATOC for localization of PCC. In contrast to DOTATOC, FDOPA also identified all PGL but with a limited number of patient cases.

## Background

Pheochromocytoma and paraganglioma (PPGL) are rare neuroendocrine tumors originating from chromaffin cells found either in the adrenal medulla [pheochromocytomas (PCC)] or in the sympathetic and parasympathetic ganglia [paragangliomas (PGL)] [1]. Most of these tumors secrete catecholamines, leading to an increase in plasma metanephrines and often characteristic symptoms such as headaches, palpitations, and sweating [2], and 10–17% of PPGL are metastatic [3]. Once there is a clinical and biochemical suspicion of PPGL, localization of the tumor and possible metastatic disease become necessary [2]. However, it is yet to be determined which imaging modality has the highest diagnostic accuracy.

Conventional morphological imaging such as computerized tomography (CT) has low specificity for PPGL, since it cannot distinguish PCC from other types of adrenal lesions or determine whether extraadrenal lesions are PGL, metastases, or of non-PPGL origin. Several functional imaging techniques have, on the other hand, demonstrated high accuracy in identifying both PPGL and metastatic lesions [4].

Prior research has shown that both [^18^F]FDOPA PET (FDOPA) (metabolic precursor for catecholamine synthesis) and [^68^Ga]DOTATOC PET (DOTATOC) (conjugated somatostatin receptor binding peptide) are valuable for identifying PPGL [4]. FDOPA seems to be the best modality for detection of pheochromocytoma because of its high sensitivity and specificity and because of its relatively low physiological tracer uptake in the normal adrenal gland [5–7]. Thus, the 2019 guideline from EANM recommends FDOPA as first choice modality of PPGL imaging in sporadic and inherited PCC [4]. DOTATOC, on the other hand, is considered more sensitive in detecting extraadrenal and metastatic disease [4, 8]. However, few studies have compared the two modalities directly.

Therefore, we conducted a retrospective single-center study comparing the diagnostic value of FDOPA and DOTATOC in the workup of patients with clinical and biochemical suspicion of PPGL. We also examined the diagnostic value in patients with previous PPGL under suspicion of recurrence (either local, new PPGL, or metastatic disease).

## Materials and methods

### Patients

The study included all patients referred for PPGL functional imaging from the Department of Endocrinology and Internal Medicine to the Department of Nuclear Medicine and PET Centre, Aarhus University Hospital in the period January 10, 2018, to May 3, 2022. All patients were referred on a clinical and biochemical suspicion of PPGL (i.e., with elevated metanephrines on consecutive measurements and either an adrenal incidentaloma or a relevant clinical genetic predisposition). Five patients only had measurements of methoxynoradrenalin. Elevated metanephrines were calculated as an index according to the upper normal limit [9], i.e., a value of 2.01 and an upper limit of 0.45 give a calculated index of 3.47. All patients with normal metanephrines were given a basic value of 1.

We also included patients referred on the suspicion of PPGL recurrence (local recurrence, new PPGL, or metastases). These patients were analyzed separately.

Each patient underwent both a DOTATOC and a FDOPA scan for diagnostic purposes. The PET scans were on average performed within a span of 9 days (minimum 1 day in between).

### Ethics

The institutional review board at Aarhus University Hospital granted access to patient files. According to Danish legislation, individual patients’ consent was waived due to the retrospective nature of the study. All methods were carried out in accordance with relevant guidelines and regulations.

### Tracer production

FDOPA was produced using a 16.8-MeV PETtrace cyclotron (GE Medical Systems, Uppsala, Sweden) and according to a standard procedure [10].

DOTATOC was produced by standard procedures using a registered product (edotreotide, SomaKIT TOC), as described by Manoharan et al. [11].

### PET recordings

The subjects were scanned on three scanner models: (1) A Siemens Biograph Truepoint TrueV (2018–2019) (*N* = 18), (2) Siemens Biograph Vision 600 (2019–2022) (*N* = 63) and (3) GE Healthcare Discovery MI scanner (2019–2022) (*N* = 32). All scanners were cross-calibrated using an ^18^FDG phantom.

Truepoint emission data were corrected for attenuation, based on a low-dose CT (CARE Dose 4D, 50 mAs, 120 kV). PET reconstruction parameters were TrueX, 4 iterations, 21 subsets, 336 matrix, 3-mm Gaussian postfilter, voxel size 2.0^3^ mm^3^ and a central spatial resolution around 5-mm FWHM.

Vision emission data were corrected for attenuation based on a low-dose CT (CARE Dose 4D, 25 mAs, 120 kV, CARE kV). PET reconstruction parameters were TrueX + TOF, 4 iterations, 5 subsets, 440 matrix, 2-mm Gaussian postfilter, voxel size 1.65^3^ mm^3^ and a central spatial resolution around 3.5-mm FWHM.

Discovery emission data were corrected for attenuation based on a low-dose CT (Smart mA, Noise index 43, 100 kV). PET reconstruction parameters were VPFX-S, 3 iterations, 16 subsets, 384 matrix, 3-mm Gaussian postfilter, voxel size 1.8 × 1.8 × 2.8 mm^3^ and a central spatial resolution around 3.9-mm FWHM.

A low-dose CT without contrast was performed for attenuation and coregistration purposes. However, if a diagnostic CT scan had not been performed within 1 month before the first PET scan, a contrast-enhanced CT scan (arterial phase of thorax, abdomen and pelvis and a portal phase of upper abdomen) was performed according to local procedures for neuroendocrine tumor-imaging.

### PET/CT scans

All the scans were re-evaluated by two experienced nuclear medicine specialists blinded to the clinical outcome, with access to the same information as present at the time of the scans (e.g., previous PPGL, result of previous CT scans, and if metastatic disease was suspected). The two scans were evaluated consecutively in the same session in random order.

The results of PET scans were recorded for both adrenal and extraadrenal locations. The findings in the adrenals were registered whether there was a PCC present and if so, the side location was noted to do a lesion-based comparison to the clinical diagnosis. The extraadrenal lesions were recorded with location and number and assessed as single, multiple, or metastatic PGL.

### [^18^F]FDOPA

The subjects were told not to take drugs or food for 4 h before the scan but were free to drink water. No patients were premedicated with carbidopa. Sixty minutes after intravenous injection of 400 MBq [^18^F]FDOPA patients were placed in a supine position and scanned from top of the skull to mid-thigh.

The co-registered FDOPA PET/low-dose CT images were displayed together with a contrast-enhanced CT. FDOPA uptake in the adrenal glands was considered pathological if there was asymmetric uptake correlated to a focal lesion. If there were no focal lesions in any of the glands, symmetrical uptake was considered pathological if noticeably more intense than the liver. FDOPA uptake outside the adrenal glands was considered pathological if there was any uptake beyond the surrounding tissue in a non-physiologic distribution. Maximum standardized uptake value (SUV_max_) was measured for each lesion.

### [^68^Ga]DOTATOC

The were no special preparations for the subjects before the scan. Ninety minutes after injection of [^68^Ga]DOTATOC, 2.5 MBq/kg, a minimum of 100 MBq and a maximum of 250 MBq, patients were placed in a supine position and scanned from skull to mid-thigh.

When interpreting DOTATOC PET, two conditions were considered, namely the absolute uptake in an adrenal lesion and, because the physiological uptake is high in the adrenals, also the difference in uptake between the two adrenals (ie the healthy one and the diseased). If there were lesions in both adrenal glands, we only looked at the absolute uptake. The absolute uptake had to be above liver level, i.e., at least Krenning 3 (modified Krenning scale: 0 = no lesions, 1 = weak uptake, considerable uptake below liver level; 2 = moderate uptake, less than or equal to the liver; 3 = intense uptake above the liver and below the spleen; and 4 = excessive uptake above the spleen). Outside the adrenal glands, any focal lesions with increased DOTATOC uptake were considered pathologic if assessed as Krenning 2 or higher. We defined negative DOTATOC scans as Krenning 2 scores or below, without difference in uptake in the two adrenal glands.

### Clinical diagnosis as gold standard

The accuracy of the patient- and lesion-based PET diagnoses was analyzed using the final clinical diagnosis as gold standard. The final clinical diagnosis was primarily based on pathological examinations. In the few instances where no pathological examination was available (eg. if patients did not undergo surgery), the clinical diagnosis was based on an overall clinical assessment consisting of a combination of the available imaging, laboratory tests, and (when applicable) the follow-up data.

### Statistics

We used a nonparametric test (Mann–Whitney, two-tailed) to compare differences between groups of patients, with probability values of less than 0.05 as the threshold of statistical significance. A nonparametric test was used to test correlation between XY-pairs (Spearman r test with computation of nonparametric Spearman correlations, two-tailed, confidence interval 95%).

To compare the two scanning modalities for sensitivity and specificity, a McNemar’s test was used.

## Results

### Study population

In total, 113 patients were included in the study of whom 97 were referred on the suspicion of primary PPGL and 16 on suspicion of recurrence. After diagnostic workup, 57 (59%) of 97 newly referred patients were diagnosed with PPGL. Age, sex, genetic status, and PPGL types are summarized in Table 1. Eleven (69%) of the 16 with previous PPGL were confirmed to have PPGL.

**Table 1:** Patients suspected of primary PPGL

	PPGL confirmed	PPGL refuted
Individuals, *n* = 97		
Age, median (range)	58 (15–86)	58 (27–88)
Female sex, *n* (%)	26 (46%)	22 (55%)
PPGL type, *n* (%)	56	40
Unilateral PCC	47 (82%)	NA
Bilateral PCC	4 (7%)	NA
Single PGL	5 (9%)	NA
Multiple PGL	(0%)	NA
Both PCC and PGL	0 (0%)	NA
Metastatic PPGL, *n* (%)*	1 (2%)	NA
Genetic status		
Pathogenic variant**	16	10
No pathogenic variant	37	2
Not genetically tested	4	28
Biochemical values, mean (range)		
Methoxyadrenaline	5.3 (1.0–35.3)	1.3 (1.0–4.3)
Methoxynoradrenalines	7.4 (1.0–43.3)	1.2 (1.0–2.8)

*Bladder wall metastatic lesion from PGL

**Genetic variants included VHL (*n* = 5), MEN2A (*n* = 14), SDHB (*n* = 3), NF1 (*n* = 4)

### Diagnostic performance of FDOPA and DOTATOC for pheochromocytomas (primary PPGL)

FDOPA had higher sensitivity for detection of PCC compared to DOTATOC (patient-based: 100 vs. 49%, *p* < 0.001; lesion-based: 100 vs. 49% *p* < 0.001), whereas the specificity was similar for the two modalities (patient-based 95 vs. 98% *p* = 1.00; lesion-based: 98 vs. 99%, *p* = 0.62) (Table 2). In addition, FDOPA had a higher negative predictive value and diagnostic accuracy compared to DOTATOC (both *p* < 0.001).

**Table 2:** Diagnostic performance of [^18^F]FDOPA and [^68^Ga]-DOTATOC PET in primary adrenal disease (patient- and lesion-based analysis)

	[^18^F]FDOPA	[^68^Ga]DOTATOC
Patient-based (*N* = 97)		
Sensitivity^a^	51/51 (100%)	25/51 (49%)
Specificity	38/40 (95%)	39/40 (98%)
PPV	51/53 (96%)	25/26 (96%)
NPV^a^	40/40 (100%)	39/65 (63%)
Diagnostic accuracy^a^	90/92 (98%)	64/91 (70%)
Lesion-based (*N* = 194)		
Sensitivity^a^	55/55 (100%)	27/55 (49%)
Specificity	136/139 (98%)	138/139 (99%)
PPV	55/58 (95%)	27/28 (96%)
NPV^a^	136/136 (100%)	138/166 (83%)
Diagnostic accuracy^a^	191/194 (98%)	165/194 (85%)

*PPV* positive predictive value, *NPV* negative predictive value

^**a**^Mean value of patients with PCC is significantly different compared with mean of patients without PCC (*P* < 0.001)

### FDOPA SUV_max_ in adrenal lesions related to clinical diagnosis (primary PPGL)

**Fig. 1:**
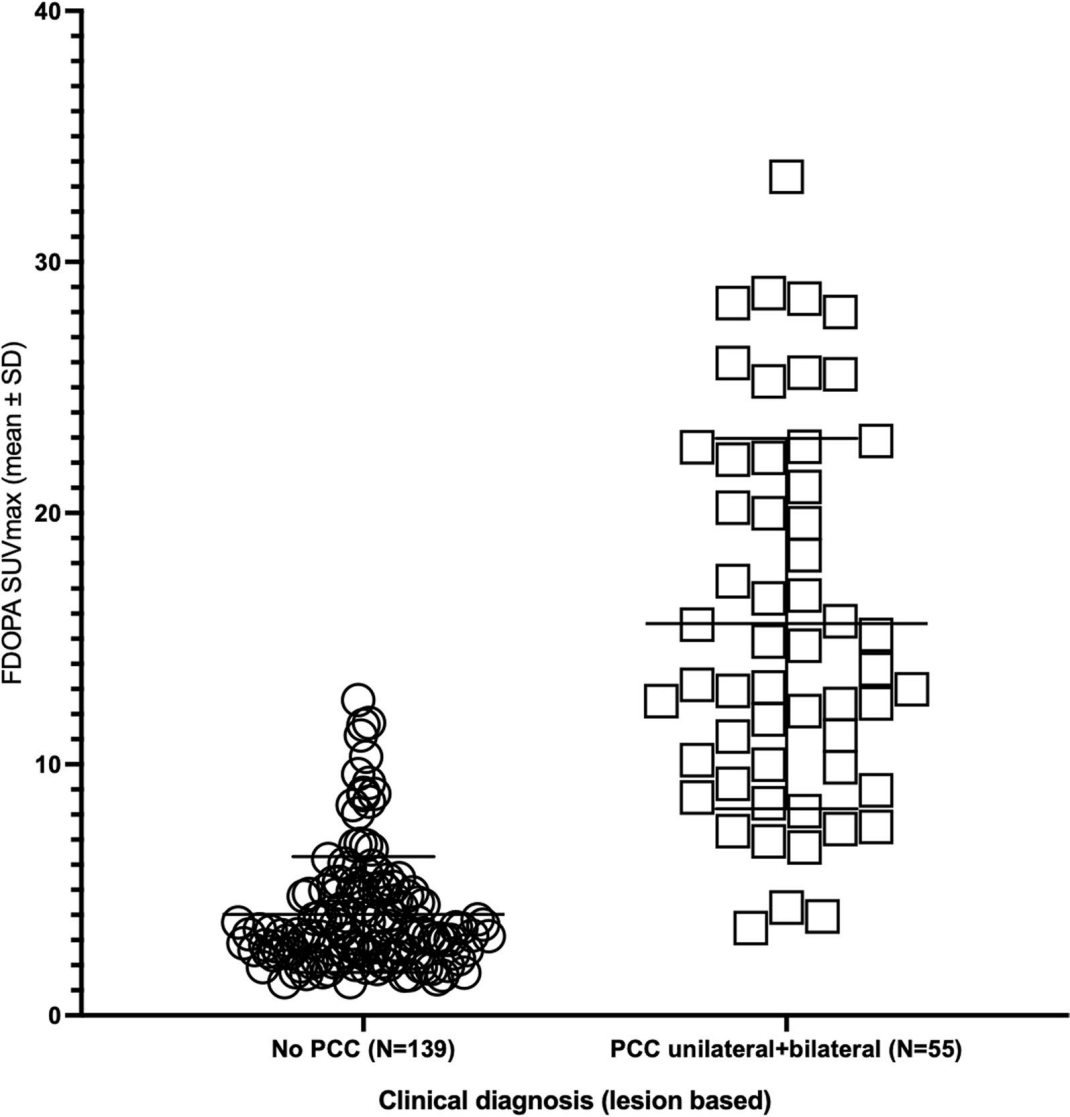
Clinical diagnosis related to lesion-based [^18^F]FDOPA PET SUV_max_ values in patients suspected of primary PPGL. Individual values of [^18^F]FDOPA SUV_max_ in adrenals of patients without pheochromocytoma (circle) (‘No PCC’) and in adrenals of patients with unilateral and bilateral pheochromocytoma (square) (‘PCC, unilateral + bilateral’). Mean  ± SD is indicated for each group with horisontal lines

Mean FDOPA SUV_max_ differed between patients with PCC (unilateral and bilateral) and patients without PCC (*p* < 0.001). FDOPA SUV_max_ was 4.0 ± 2.3 (mean ± SD) (range 1.3 to 12.6) in adrenal glands in patients without PCC, 15.6 ± 7.3 (range 3.5 to 28.8) in adrenal lesions in patients with unilateral PCC, and 17.8 ± 8.9 (range 9.2 to 33.4) in adrenal lesions in patients with bilateral PCC (Fig. 1).

**Fig. 2:**
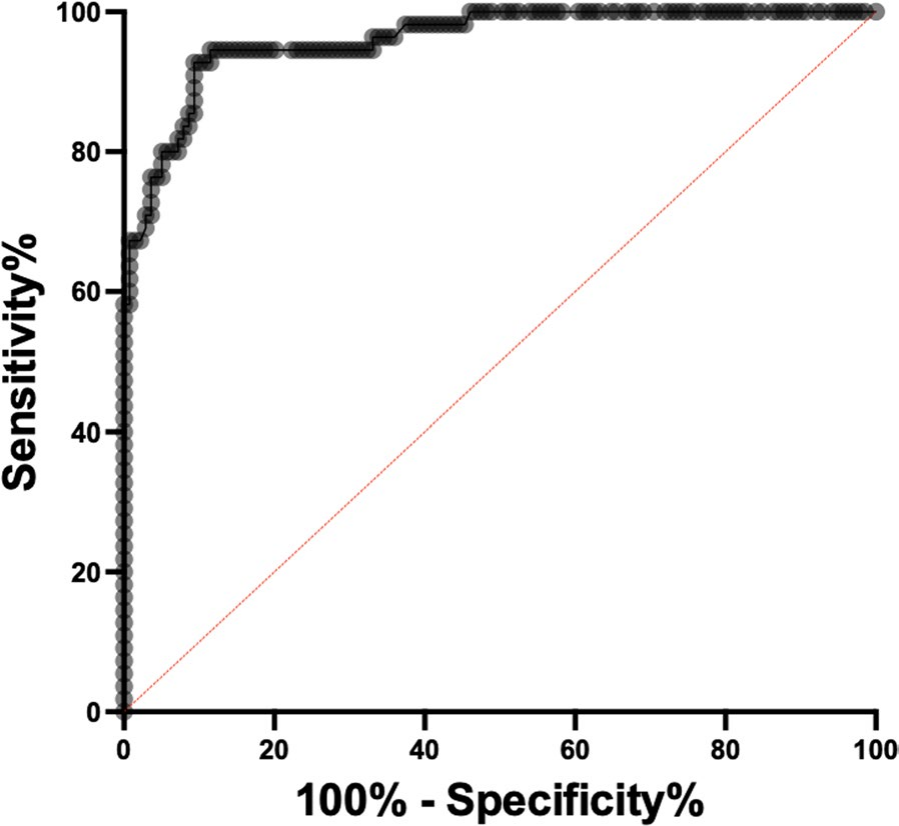
ROC of [^18^F]FDOPA PET SUVmax values in adrenals of patients suspected of primary PPGL

A receiver operating characteristic (ROC) analysis was used to evaluate sensitivity and specificity based on FDOPA SUV_max_ values (AUC: 0.96, *p* < 0.001) (Fig. 2). The optimal cutoff of SUV_max_ was resulting in 93% sensitivity and 91% specificity for diagnosing pheochromocytoma.

**Fig. 3:**
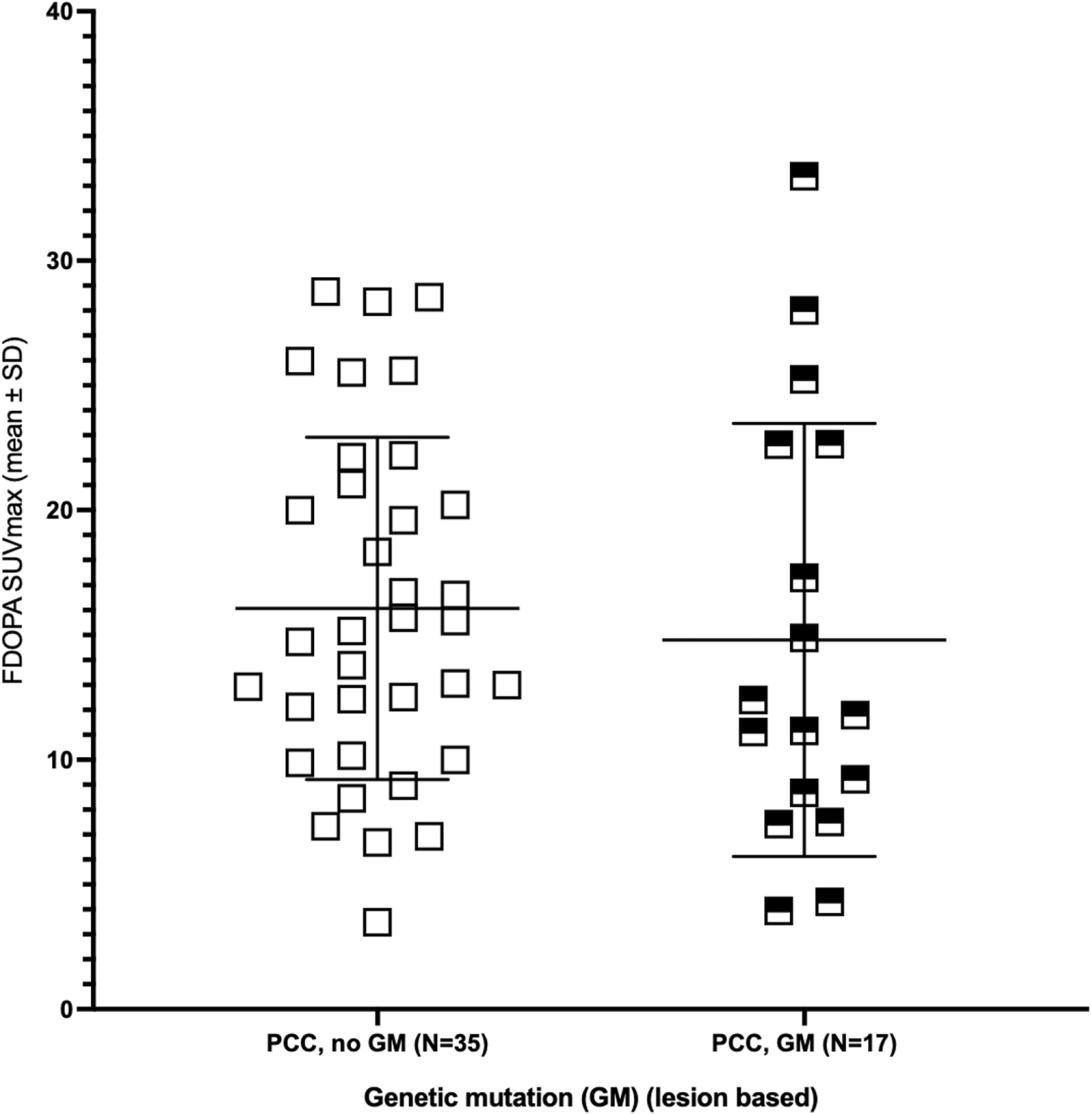
Relation of mutation status to lesion-based [^18^F]FDOPA PET SUV_max_ in adrenals of patients suspected of primary PPGL**.** Individual values of FDOPA SUV_max_ for patients with pheochromocytoma and no genetic mutation (all patients had unilateral phaochromocytoma) (square) (‘PCC, no GM’) (N = 35), and for patients with unilateral (N = 9) or bilateral pheochromocytoma (N = 4) and a genetic mutation, ie. 17 observations (filled open square) (‘PCC, GM’). Mean ± SD is indicated for each group with horisontal lines

No difference was observed in FDOPA SUV_max_ in adrenals of PCC patients with or without a pathogenic genetic variant (14.8 ± 8.4 vs. 16.1 ± 6.8, *p* = 0.42) (Fig. 3).

**Fig. 4:**
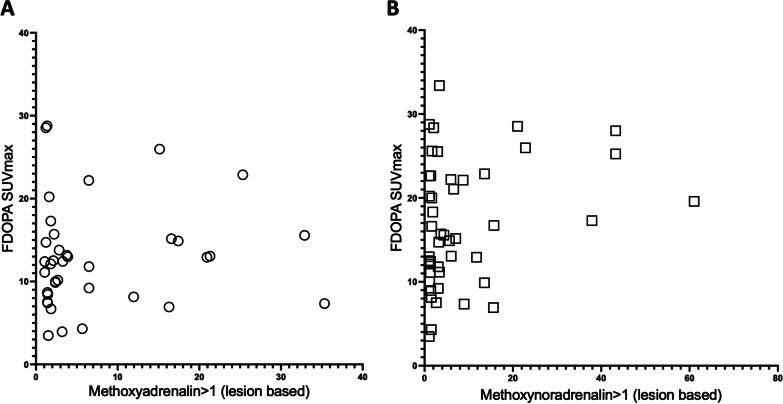
Elevated plasma methoxynoradrenalin and adrenalin versus [^18^F]FDOPA PET SUV_max_ in patients suspected of primary PPGL. Individual values of adrenal FDOPA SUVmax for patients with pheochromocytoma versus calculated methoxyadrenalin (N = 38) (circle) (calculated quotient normalized to age and sex) (**A**) and calculated methoxynoradrenalin (N = 47) (square) (calculated quotient normalized to age and sex) (**B**), respectively

In patients with PCC, adrenal FDOPA SUV_max_ showed no correlation to the plasma level of methoxyadrenaline or methoxynoradrenaline (including calculated pathologic values above 1) (Fig. 4).

**Fig. 5:**
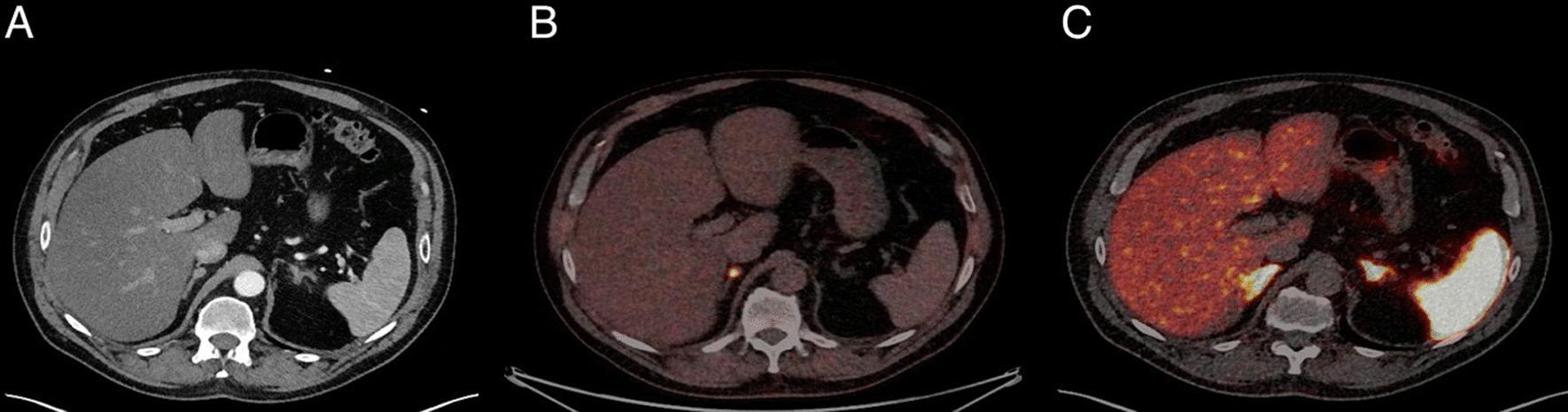
Clinical case presenting a patient suspicious for PPGL. The patient had a history of hypertension. Preoperative normetanephrines were 1.93 and 1.59 (< 1.07 nmol/L) and metanephrines 0.23 and 0.23 (< 0.45 nmol/L). Pathology confirmed a 15-mm pheochromocytoma with a PASS score of 7. The images show transaxial slides of the adrenal glands on a contrast-enhanced CT (left, image **A**), superimposed on a contrast-enhanced CT for FDOPA PET/CT (middle, image **B**) and DOTATOC PET/CT (right, image **C**). Image B shows high, FDOPA uptake in the right adrenal gland correlating to a 15-mm nodular, contrast-enhanced structure (not hypervascular). No FDOPA uptake in the left adrenal gland and no CT correlate. Image **C** shows the high physiologic uptake of DOTATOC in both adrenal glands, as well as physiological uptake in the liver and spleen. Blood pressure and plasma metanephrines were normalized after surgical resection

Figure 5 shows a clinical case of a patient suspected of primary PPGL. This case illustrates the advantage of using FDOPA for detecting small structures in the adrenal glands due to the low physiological uptake in the normal adrenal glands as opposed to the high physiological uptake with DOTATOC.

**Fig. 6:**
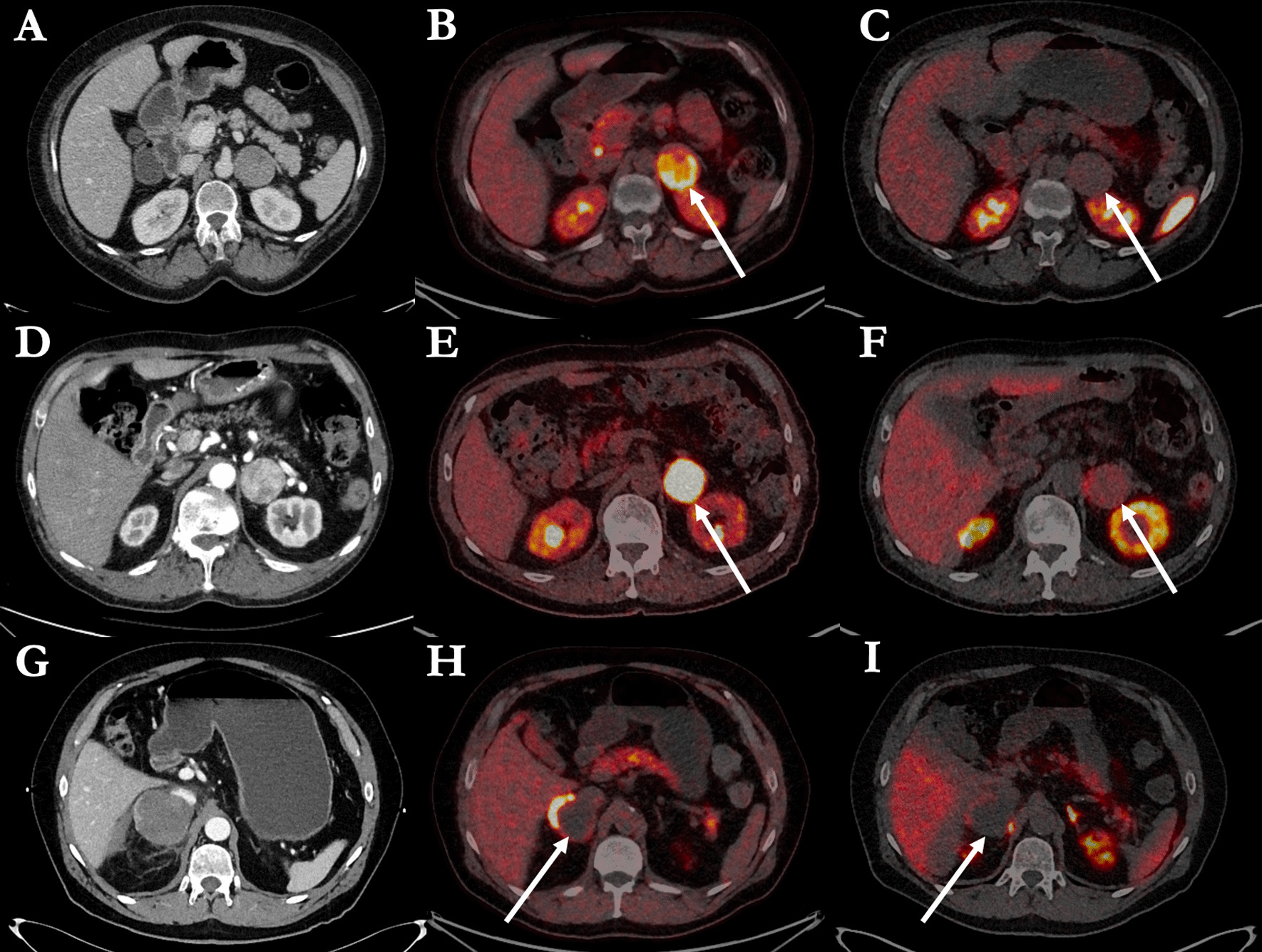
Clinical cases presenting large size pheochromocytomas in three patients suspicious for PPGL. Patient 1 (image **A**, **B**, **C**). 51-year-old women with a history of hypertension and paroxysmal palpitations, sweating and headache. Preoperative plasma normetanephrines were 13.30 and 12.30 (< 0.98 nmol/L) and metanephrines 1.07 and 0.99 (< 0.45 nmol/L). Pathology confirmed a 66-mm pheochromocytoma with a PASS score of 10*.* Symptoms, blood pressure and plasma metanephrines were normalized after surgical resection. *Patient 2* (image **D**, **E**, **F**). 72-year-old male with a history of hypertension, weight loss, discomfort, and paroxysmal palpitations. Preoperative plasma normetanephrines were 6.36 and 7.68 (< 1.07 nmol/L) and metanephrines 6.55 and 7.46 (< 0.45 nmol/L). Pathology confirmed a 38-mm pheochromocytoma with a PASS score of 7*.* Symptoms, blood pressure and plasma metanephrines were normalized after surgical resection. *Patient 3* (image **G**, **H**, **I**). 69-year-old male with a 10-year history of hypertension. Within the last year paroxysmal palpitations, nausea, and sweating. Preoperative plasma normetanephrines were 4.70 and 4.98 (< 1.07 nmol/L) and metanephrines 14.80 and 12.90 (< 0.45 nmol/L). Pathology confirmed a 45-mm pheochromocytoma with a PASS score of 9*.* Symptoms, blood pressure, and plasma metanephrines were normalized after surgical resection. The images show transaxial slides of the adrenal glands on a contrast-enhanced CT (left column, image **A**, **D** and **G**), FDOPA PET/CT (middle column, image **B**, **E**, and **H**) and DOTATOC PET/CT (right column, panel **C**, **F**, and **I**). Images B, E, and H show high FDOPA uptake within (**B**, **E**) or in the periphery (**H**) of nodular structures measuring 45, 58, and 66 mm, respectively. Images **C**, **F**, and **I** show uptake of DOTATOC below the physiological uptake of the adjacent and contralateral adrenal gland in each of the three patients. Patient 3 had a tumor in the right adrenal gland with central bleeding and a contrast-enhanced peripheral zone with high FDOPA uptake and no DOTATOC uptake besides physiological uptake medial to the tumor (image **G**, **H**, **I**)

Figure 6 shows three clinical cases of patients suspected of primary PPGL. These cases illustrate the advantage of using FDOPA, not only in patients with small structures in the adrenal glands, but also in patients with large size PC. All cases show FDOPA-positive lesions with no correlating DOTATOC uptake (below physiological uptake in the adherent and contralateral adrenal gland).

### Diagnostic performance of FDOPA and DOTATOC for paragangliomas (primary PPGL)

Six patients had paragangliomas, 5 single PGL, and 1 metastatic PGL. All were detected by FDOPA, whereas DOTATOC failed to detect three paragangliomas.

### Results for patients with previous PPGL (recurrent PPGL)

Of the 16 patients referred for suspected recurrent disease, 2 had local recurrence, 4 had single PGL, and 5 had metastatic PCC. In 5 patients, the diagnosis was refuted (Table 3).

**Table 3:** Head-to-head comparison of ^18^F-FDOPA and ^68^Ga-DOTATOC in recurrent detection

	^18^F-FDOPA	^68^Ga-DOTATOC
Patient level		
PGL incl. glomus tumor	4/4 (100%)	2/4 (50%)
Local recurrence	2/2 (100%)	0/2 (0%)
mPHEO	5/5 (100%)^a^	4/5 (80%)^a^
Lesion level		
PGL incl. glomus tumor	4/4 (100%)	2/4 (50%)
Local recurrence	2/2 (100%)	0/2 (0%)
mPHEO	22/27 (79%)^a^	23/27 (82%)^a^

*PGL* paraganglioma, *mPHEO* metastatic pheochromocytoma

^a^One patient had 3 FDOPA lesions not detectable on the DOTATOC scan, and 3 lesions on the DOTATOC scan not detectable on the FDOPA scan

All 11 patients with recurrent disease were detected by DOPA, while 5 patients had no detectable disease on DOTATOC. In two patients with metastatic PCC, the number of lesions was different; in one patient, there were more lesions on DOPA, in the other there were more lesions on DOTATOC.

## Discussion

The present retrospective, single-center study is the largest study to date to present a head-to-head comparison of the diagnostic performance of FDOPA and DOTATOC in patients suspected of PPGL. The study clearly shows that [^18^F]FDOPA PET is superior to [^68^Ga]DOTATOC PET in diagnostic imaging of pheochromocytoma both in terms of confirmation of the diagnosis and in determining the localization. In addition, FDOPA seems to be as sensitive as DOTATOC in detecting extraadrenal PPGL, although the number of patients was limited for this evaluation.

Only two other studies with a very limited number of participants compare the performance of FDOPA and [^68^Ga]-conjugated somatostatin receptor binding peptides in the detection of PCC. In a study by Archier and colleagues [12], which included 10 patients with primary and secondary PCC, FDOPA was more sensitive than [^68^Ga]DOTATATE. In this study, FDOPA detected 7 out of 7 primary PCC, while [^68^Ga]DOTATATE found 6 out of 7. With regard to recurrent PCC, FDOPA detected 4 lesions out of 4, and [^68^Ga]DOTATATE 2 out of 4 lesions. A prospective study by Jha et al. [13] included 14 patients with histologically confirmed sporadic, primary PCC. The positivity rate for PCC was 100.0% (11/11) for FDOPA compared to 78.6% (11/14) for [^68^Ga]DOTATATE PET/CT. However, these analyses were obviously weakened by the limited number of participants, which hindered valid statistical comparison of modalities.

Other studies of the performance of FDOPA, report similar results as in this present study with a high sensitivity of FDOPA in diagnosis of PCC [5–7, 12]. Hoegerle et al. [7] reported a sensitivity of 100% in a group of 14 patients with 17 PCC lesions. Imani et al. [5] found 7 out of 8 PCC in a group of 25 patients. Luster et al. [6] located 15 out of 15 PCC in a group of 25 patients.

Another study dealing with the performance of [^68^Ga]DOTATATE alone [14] shows a high sensitivity of 88% in localizing primary pheochromocytomas in a group of 46 patients, thus performing considerably better than in our study, where we found only 49% in a group of 51 patients.

One contributing factor to the high sensitivity of FDOPA in PCC is likely to the low physiological uptake in normal adrenal glands. In contrast, DOTATOC has very high physiological uptake in normal glands making smaller lesions difficult to detect.

The current study has a relatively small number of patients suspected of recurrent disease (*N* = 16), of which extraadrenal disease was found in 11 patients (Table 3). Surprisingly, FDOPA appeared to perform equally as good as DOTATOC in detecting extraadrenal disease in both paraganglioma and in recurrent and disseminated pheochromocytoma, although none of the values were statistically significantly different (data not shown). This was against our hypothesis and the current recommendations which favor DOTATOC over FDOPA in extraadrenal disease [4, 8].

Like in other similar FDOPA studies [15, 16], we measured the SUV_max_ in all adrenal glands and as expected there was significantly higher uptake in diseased glands compared to healthy glands. Nevertheless, there was a large overlap in adrenal SUV_max_ values between the group of patients with PCC and without PCC, and furthermore, there was a wide range of uptake in the diseased glands (Fig. 1). The highest SUV_max_ value in a normal gland was 12.6 and the lowest value in pathologic gland with pheochromocytoma was 3.5.A ROC analysis found an optimal FDOPA SUV_max_ cutoff value of 7, resulting in 93% sensitivity and 91% specificity for diagnosing adrenal PCC (Fig. 2).

However, because of the large overlap between SUV_max_ in normal and diseased adrenal glands cannot be determined by a simple cutoff value of FDOPA SUVmax. The interpretation should instead be based on an overall assessment including whether a morphological change is present, its characteristic structure, the degree of SUV uptake and a comparison with the SUV of the contralateral adrenal gland. The diagnosis of bilateral adrenal disease is primarily based on a very high level of FDOPA uptake, since in most cases there were no measurable lesions in CT, probably due to small lesions.

In accordance with other studies [5, 17], we found no correlation between FDOPA SUV_max_ values in PCC’s and the plasma level of methoxyadrenaline or methoxynoradrenaline. This supports that an increased FDOPA uptake is a sensitive indicator of pheochromocytoma even when plasma levels of metanephrines are only slightly elevated.

We acknowledge several limitations to our study. The retrospective design is a limitation, but we re-evaluated all scans been given only the same information as we would have in a prospective design. Another limitation is the small number of patients with extraadrenal disease involvement, and so these results should be interpreted with caution. However, the present study is still comparable to other studies, presenting more patients than any other study.

Another limitation is the lack of histological proof in some of the patients. In addition, the low frequency of genetic testing is also a limitation of the current study. It has been suggested that FDOPA performs poorly in detecting PPGL in patients with SDHX mutations (18). Our study could not address this, since we only included 3 patients with SDHB mutations, none of which had PPGL.

## Conclusions

In conclusion, FDOPA is a very sensitive tool and superior to DOTATOC in detection and localization of PGL. In addition, FDOPA seems to be as sensitive as DOTATOC in imaging extraadrenal disease. If available, we suggest that FDOPA should be the initial localization modality for PPGL.

## Data Availability

The datasets used and analyzed during the current study are available from the corresponding author on reasonable request.

## Abbreviations

PPGL: Pheochromocytoma and paraganglioma
PGL: Paragangliomas
PCC: Pheochromocytomas
FDOPA: [^18^F]FDOPA PET
DOTATOC: [^68^Ga]DOTATOC PET
